# Food Intakes by Preschool Children in Flanders Compared with Dietary Guidelines

**DOI:** 10.3390/ijerph5040243

**Published:** 2008-12-08

**Authors:** Inge Huybrechts, Christophe Matthys, Carine Vereecken, Lea Maes, Elisabeth HM Temme, Herman Van Oyen, Guy De Backer, Stefaan De Henauw

**Affiliations:** 1 Department of Public Health, Ghent University, University Hospital 2BlokA, De Pintelaan 185, 9000 Gent, Belgium; 2 Unit of Epidemiology, Scientific Institute of Public Health, Juliette Wytsmanstraat 14, 1050 Brussel, Belgium; 3 Department of Health Sciences, Vesalius, Hogeschool Gent, Keramiekstraat 80, 9000 Gent, Belgium

**Keywords:** Food intake, Recommendations, Preschool, Children, Belgium

## Abstract

The objective of this study was to compare food group intakes among preschool children with food-based dietary guidelines (FBDG) and to determine the proportion of children meeting these guidelines. Food consumption of preschool children (2.5–6.5 years) living in Flanders (Belgium) were assessed in a cross-sectional study, using proxy reported 3d estimated dietary records (EDR) (*n* 696). Statistical modelling was used to account for within-individual variation in the 3d EDR. Mean daily intakes of most food groups (beverages, vegetables, fruit and milk) were below the minimum recommendations. Only ‘grains and potatoes’ and ‘meat products’ were in line with the recommendations and ‘bread and cereals’ showed borderline intakes. Mean intakes of energy-dense and low-nutritious foods, which are discouraged within a healthy diet (like snacks and sugared drinks), were high. Furthermore, the percentage of children complying with the different food-based dietary guidelines was for most food groups extremely low (ranging from approximately 4% for fluid and vegetable intakes up to 99% for potato intakes). Boys had in general higher mean intakes of the recommended food groups. In conclusion, preschool children in Flanders follow eating patterns that do not meet Flemish FBDG. Although the impact of these eating habits on preschooler’s current and future health should be further investigated, it is clear that nutrition education and intervention are needed among preschool children and their parents in Flanders.

## 1. Introduction

Although good nutrition is important during the whole life course, it is especially important during the first years of life, since these are the most crucial years for normal physical and mental development. In young children, dietary intake is not only linked to growth, development and nutrition-related diseases (such as deficiencies and toxicities), but also to risk factors for chronic diseases such as obesity, increased cholesterol levels and hypertension [1]. In these first years of life children acquire many of the physical attributes and the social and psychological structures for life and learning [2]. Because unhealthy lifestyle patterns might continue into adulthood [3], it is important to strive as early in life as possible for a high-quality diet with optimal levels of food and nutrients to help maintain optimal health.

The role of certain nutrients and foods in the prevention of chronic diseases like cardiovascular disease (CVD) and some cancers has been highlighted by a large number of studies [4, 5]. Specifically, diets with high fruit and vegetable intakes have been shown being associated with a lower risk of mortality [6] and of suffering cancer [5] or CVD [7]. Because of this major role of nutrition in the prevention of diseases as well as in growth and development, a series of recommended dietary allowances (RDA) [8, 9] ranging from infancy until old age, have been set both at a national and international level. In order to motivate the public to meet such recommendations, Food-Based Dietary Guidelines (FBDG) were developed based on these RDA, though expressing recommended daily intakes at the food group level, have been drawn up in different countries [10, 11].

In Flanders, in 1997 dietary guidelines for the public have been visualized in the form of a triangle, named the Flemish ‘food triangle’ [10]. More recently specific FBDG for preschool children, also visualized as a food triangle (Appendix 1) [10], were developed in addition and are currently being used in preschool nutrition programs. This food triangle promotes a healthy daily diet, based on the consumption of seven major food groups (non sugared beverages; bread, cereals, potatoes, and grains; vegetables; fruit; milk products & calcium-enriched soy products; meat, fish, eggs and meat substitutes; fat & oils), including recommended daily portion sizes (expressed in g/d) for these food groups. Furthermore, it proposes large dietary variety and it recommends children to be physically active. An additional food guide attached to this food triangle is giving suggestions for healthy food choices within each food group. Evaluating diets by comparing these FBDG with actual food intakes complements traditional research, which focused mainly on energy and nutrient dietary consumptions [12].

Since dietary habits start being formed in early childhood it is important to ascertain the degree to which dietary habits in children comply with the pertinent recommendations. Unfortunately, studies conducted on young children’s eating habits in Flanders are scarce and the few studies conducted were local or regional in scope. Therefore, in an attempt to bridge this gap in available descriptive data, a population-based survey has recently been carried out in preschool children in Flanders, aimed at estimating nutrient and food intakes. The results on usual energy and nutrient intakes from this study have been reported and interpreted in the perspective of existing national and international nutrient recommendations in a previous paper [13]. Although this comparison with nutrient RDA is extremely relevant, a complementary approach by assessing compliance with FBDG is necessary in order to obtain an overall view of preschool children’s dietary problems. The results of this study allow us to determine whether food triangle recommendations are being met among preschool children (2.5 – 6.5 years) in Flanders and whether gender and age differences exist in food group intakes.

## 2. Subjects and methods

### 2.1. Study population and design

Data used for these analyses derived from a cross-sectional study in preschool children (2.5 – 6.5 years) in Flanders, using a multistage clustered sampling design, with schools as primary sampling units and classes as secondary sampling units. The study design and methodology of this study have been described in more detail elsewhere [14]. The method used to collect detailed information about the children’s food intake was 3d Estimated Dietary Records (EDR). The parents received written instructions for the recording of the foods and drinks consumed by their child. The schoolteachers received written and oral instructions for the recording of foods and drinks consumed during school days (e.g. snacking and lunches). Teachers had to report this information about what the children consumed at school to the parents/proxy so that they could include it in the diaries. In these structured EDR, days were subdivided into six eating occasions: breakfast, morning snacks, lunch, afternoon snacks, dinner, and evening snacks. Detailed information on the type (including brand names) and portion size of the foods consumed was collected using an open entry format. On a separate sheet, parents were invited to give details on recipes, ingredients, cooking methods, etc. After collection, the EDR was checked on quality and completeness. Only good quality EDR, including three completed record days and containing sufficiently detailed descriptions of the food products and portion sizes consumed, were included in the analysis. However, use of standard portion sizes was inevitable for some food products (e.g. French fries, mixed recipes, vegetables…), for which portions sizes were difficult to describe/estimate by the respondent (parents or another proxy). Two dieticians, with a longstanding experience in nutritional epidemiological fieldwork, carried out the exclusion procedure of the EDR. This quality control procedure has been described elsewhere [14]. The fieldwork of this study was carried out from October 2002 until February 2003 and the Ethical Committee of the Ghent University Hospital (Belgium) granted ethical approval for the study. In total 2095 children (spread over 43 nursery schools) were asked to complete the 3d EDR.

### 2.2. Comparison with food based dietary guidelines

All foods reported were categorized into food groups, in order to allow comparison with the Flemish FBDG. Mixed dishes reported in the EDR were first disaggregated into their ingredients. Thereafter all foods and ingredients reported in the EDR, were also assigned to their appropriate triangle group. It is noteworthy that the complex food mixture pizza has not been broken down into its constituent components as the ingredients of pizza were seldom described in sufficient detail in the diaries. Therefore, pizza was categorized in the food group salty snacks.

Like the guidelines given in the triangle, the quantity of all the food groups consumed was expressed in g/d (or mL/d for fluids). To allow comparison of portion sizes reported in the diet records with the daily portion sizes recommended in the FBDG, the portion size of some reported foods had to be converted in an equivalent of another food using conversion factors proposed in the food guide (Appendix 1) [10]. For rice, for instance, its portion size had to be converted to an equivalent portion size of potatoes as the guidelines in the food triangle are expressed in g potatoes/d. Even though foods like bread and potatoes both belong to the food group ‘cereals and potatoes’, this food group has been split into two different subgroups, ‘bread and other bread products’ and ‘potatoes and grains’, that correspond to two different recommended daily portion sizes.

As mentioned before, the triangle recommends a daily intake for each food group (non sugared beverages; bread, cereals, potatoes, and grains; vegetables; fruit; milk products & calcium-enriched soy products; meat, fish, eggs and meat substitutes; fat & oils) in order to cover the varying nutrient needs in the population. The tip of the triangle is separated from the rest of the triangle and is called the ‘restgroup’. This restgroup represents a group of non-essential food items and drinks that are discouraged within a healthy diet, often because of their high energy and/or low nutrient density.

The different food groups in the food triangle for preschool children (3–6 years) are the same as those in the triangle for older children and adults; however, the recommended daily portion sizes are different. Details about the food categorization used in this study are given in Appendix 2.

In the present study, usual food group intakes derived from the EDR are compared with the dietary guidelines visualized in the food triangle (Appendix 1). Though for comparison with some additional guidelines reported in the food guide, some extra analyses were executed for a few food items within some of the food groups presented in the food triangle (e.g. consumption of whole fat and low fat milk and the weekly consumption of fish products). Although children are also recommended to spend at least one h/d at moderate to vigorous physical activities or physically active games, compliance with this food triangle guideline could not be investigated since the data gathered concerning the physical activity level of the children was too limited.

### 2.3. Statistical methods

Usual food group intakes from the EDR were computed based on the recommendation of the Institute of Medicine (IOM) regarding the need to determine the distribution of usual food intakes for assessing diets of population groups in relation to the recommendations [15]. Therefore, a statistical modelling method (the NUSSER method: developed at Iowa State University) that accounts for within-individual variation in food intakes while requiring relatively few days of intake per individual was used to estimate usual dietary intakes based on the 3d EDR [16, 17]. The software used to carry out the method was the Software for Intake Distribution Estimation (C-side) [18].

This paper presents estimates of ‘usual’ food(group) intakes including the mean intakes, standard deviation (SD), median and standard error (SE) for different gender and age groups (children 2.5 – 3 and 4 – 6.5 years old will be referred to as the ‘youngest’ and ‘oldest’ age group, respectively). In addition the percentage below or above the reference values were calculated. The percentages of individuals with intakes below the food-based dietary guidelines, when using the adjusted EDR data, are estimates of the prevalence of inadequacy. Depending on the distribution characteristics, the student’s t-test or Mann-Whitney U-test was used to compare the means of two groups of children. Unless reported differently, a P-value of 0.05 was used as the threshold for significance.

## 3. Results

A total of 1,052 EDR were collected by the end of the fieldwork (Response Rate: 50%). From these 1,052 EDR, 1,026 were useful for analyses (after exclusion of low quality records). However, when excluding the children for whom only one or two days were collected or retained; only 696 children were left for the final analyses of the 3d EDR. Boys and girls were equally represented.

### 3.1. Mean daily intakes of the major food groups

Based on the results reported in Table 1, it could be concluded that mean daily intakes of almost all major food groups (beverages, vegetables, fruit, milk products, and spreadable fat) were below the minimum food-based dietary guidelines. Only for ‘grains and potatoes’ and for ‘meat products’ the mean intakes were in line with the guidelines. Mean bread and cereal intakes (e.g. breakfast cereals), reached the minimum food-based dietary guidelines for boys and was borderline for girls.

Overall, boys had higher intakes of the different food groups than girls (Table 1). In general, the oldest group of preschool children had similar to higher intakes of the different food groups than the youngest group of preschool children (except for milk products, which was higher in the youngest group of children).

When considering the mean intakes of the different subgroups from the restgroup (tip of the triangle), the estimated total daily intake of non-nutritious products that are classified in the restgroup was about 200 g/d and was higher for boys and for the oldest group of preschool children in comparison with respectively girls and younger preschoolers.

### 3.2. Percentage of children meeting major dietary guidelines

The ranges of children complying with the guidelines as described below are the minimum and maximum percentage found when considering the different age and gender groups. In Table 2, the percentages of children below and above the guidelines (thus not complying with the food-based dietary guidelines) are respectively presented for both age and gender groups separately.

In total, only 1–5% of the preschool children reached the FBDG for beverage intakes (excluding milk products & calcium-enriched soy products) of 1000 ml/d. About one third of the children was even drinking less than half (500 mL/d) of this FBDG.

Only 45–60% of the children reached the minimum FBDG for daily bread intake (including cereal products) and between 3–14% of the children had an intake above the upper level of the FBDG.

In contrast with most food groups, the percentage of children reaching the minimum FBDG for grains and potato intakes (incl. foods like rice and pasta) is high, ranging from 91 to 99% of the children. The percentage of children exceeding the upper level of the FBDG for potatoes and grain products was less than 3%.

Less than 5% of the youngest children and <20% of the oldest preschoolers reached the minimum FBDG for daily vegetable intakes. Furthermore, less than half of the children (34–45%) reached the minimum recommendation for fruit intakes.

Less than half of the children (32–55%) reached the minimum daily FBDG for the intake of milk, milk drinks and calcium-enriched soy products, while more than half of the children (51–71%) reached the minimum FBDG for cheese intake. For cheese, 18–42% of the children had even higher intakes than the maximum level of the FBDG.

About 56–83% of the children reached the minimum FBDG for daily meat, fish, egg and meat substitute intakes and 19–46% of them had an intake higher than the upper level of the FBDG.

Only 1–9% of the children reached the minimum FBDG for spreadable fat on bread.

### 3.3. Additional comparisons with dietary guidelines

Although the comparison with the extra recommendations concerning the healthier food choices and variation within the different food groups has not been presented in the tables, some important findings are reported below. First it is noteworthy that less than 30% of the children reached the weekly recommendation for fish intake (one to two times/wk 75–100 g/d). Secondly we found that 44% of our Flemish preschoolers consumed mainly white bread instead of brown (or fibre rich) bread. Thirdly our results showed that about two third of the children were using spreadable fat on their bread of whom 11% were using animal fats (butter or butter with reduced fat content). Fourthly, we found that most of the children consuming cows milk used mainly semi-skimmed milk (72%), and only 2% used skimmed milk. Although children ≥4 years old are recommended to use semi-skimmed milk, there is still more than 20% of the children ≥4 years old that are consuming whole fat milk.

## 4. Discussion

### 4.1. Main results

Several major findings have emerged from the present study. Most importantly, the overall diet of children in this sample was not compatible with that recommended by the Flemish food triangle [10]. The children in our study consumed an excessive amount of foods from the triangle ‘tip’ (like snacks and sugared drinks), while the mean daily portion consumed from all other groups, except from meat products, potatoes and grains (pasta, rice…), were borderline or far below the FBDG. Furthermore, the percentage of children complying with the different food group guidelines was for most food groups extremely low. Mainly for the intake of vegetables, fruit and fluids, the children scored far below the FBDG. Boys had in general higher mean intakes of the recommended food groups and complied therefore slightly better with the FBDG than girls did. When considering the contribution of different food items to the major food groups of the food triangle, it was clear that within these food groups, children were often making less healthy food choices. In the group of ‘cereals and bread’ for instance almost half of the children were using white bread while brown bread is recommended in the dietary guidelines attached to the food triangle. However, it should be mentioned that at this preschool age, it are still mainly the parents, the schools and peers that are responsible for those less healthy food choices.

### 4.2. Strengths and limitations of the study

Parentally reported dietary intake assessments tend to be difficult in preschool children, since these children are incapable of reporting the foods and portion sizes they consumed at school themselves. Therefore, it should be noted that the apparent low percentage of children reaching the minimum recommendations of the FBDG in the sample of preschool children could be due to a certain extend to inadequate reporting of dietary intake as well (e.g. underreporting). However, as described in more detail elsewhere [14], several steps were undertaken in this Flanders preschool dietary survey to increase the validity of the information (e.g. school staff was involved in the reporting of snacks and lunches consumed during school time and great efforts were done in order to motivate the parents). In addition, some comparisons between the results derived from a brief (forty-seven item) FFQ, completed by the parents in addition to the EDR, and the results presented from the 3d EDR showed that from both methods (which have opposite intrinsic limitation) similar conclusions could be drawn for almost all the major food groups (results not published). Furthermore, the degree of underreporting that was published previously [13], showed no children with a lower ‘energy intake/basal metabolic rate’ ratio than Goldberg’s cut-off adapted for children in the group of children younger than four years old and only <2% for children at least 4 years old (0.5% in boys, and 1.4% in girls). Therefore, bias attributable to misreporting is supposed to be only limited in this survey and cannot explain the low percentage of children reaching the minimum recommendations in our Flanders preschool dietary survey.

### 4.3. Comparison with other studies

As mentioned before, comparison with other studies in Flemish preschoolers was not possible, since this was the first diet study conducted in a representative sample of Flemish preschoolers. Nevertheless, data from the National Food Consumption survey 2004 [19], in which 2 d 24 h diet recalls are used, indicate that large proportions of the Belgian population at least fifteen years old failed to meet dietary recommendations as well. Only 7.6% reached the minimum recommendation for fruit intake (250 g/d for adults) and less than 0.1% reached the minimum recommendation for vegetable intake of 350 g/d [20].

Nevertheless, the mean and median food group intakes reported in this paper, could be compared with those reported by Alexy *et al*. from the DONALD study in Germany [21, 22]. The DONALD Study (which started in 1985) is a cohort study collecting detailed data on diet, metabolism, growth, and development from healthy subjects between infancy and adulthood (3 months – 18 years old). Parents of the children or the older subjects themselves kept 3d weighed dietary records, weighing and recording all foods and fluids consumed as well as leftovers using electronic food scales [23]. When comparing our results with those of the DONALD study (collected between 1990 and 1997), similar intakes were found for the food groups ‘bread and cereals’, ‘fruits’, and ‘vegetables’. For the food groups ‘potatoes and grain products’, ‘milk products’, ‘meat products’ and ‘beverages’ the intakes reported by our Flemish preschool children were higher than those found for preschool children in the DONALD study [21, 22]. However, for confectionery (snacks and sweet desserts) the daily intake of the children in the DONALD study was even higher (about 70 g/d) than the total intake reported in our study.

Another study in children is the enKID Study: a population-based, cross-sectional nutrition survey in Spanish children and adolescents (2–24 years) conducted between 1998 and 2000. In the enKID study, dietary assessment was completed by means of a 24h recalls and a FFQ completed in an interview with the mother or caregiver for children under thirteen years old. A second 24h recall was completed on 25% of the sample, allowing for adjustment of intakes for random intra-individual variation [24]. Although an attempt to compare our food intake results with those reported by Serra-Majem revealed higher intakes of all food groups (except milk intake), by the children participating in the enKID study [25], these higher intakes are likely to be due to the large age range (2–24 years) in the study reported by Serra-Majem *et al*. [25].

Another study in Spanish children is the cross-sectional Four Provinces study (1998–1999), which was conducted in four Spanish cities in order to assess dietary intakes among 6–7 year old schoolchildren [26, 27]. Children were selected through random cluster sampling in schools and an interviewer administered FFQ was used as dietary assessment instrument. Comparison of our results with those of Spanish children 6–7 year old who participated in the Four Provinces study, showed much higher fruit (319 g/d), vegetables (327 g/d), and dairy intakes (628 g/d) for these children in comparison with our Flemish preschoolers [26]. The mean consumption of fruits, vegetables, and dairy products were all above the recommended minimum for this Spanish age group [26]. It is interesting to see that the intake of fruits and vegetables in young Spanish children is significantly higher than in our Flemish preschoolers and German children participating in the DONALD study.

## 5. Conclusions

These results indicate the need for improvement in dietary habits among Flemish children in order to produce a healthful diet and to prevent diet-related diseases in our future adult population. Community and/or school based nutrition education programmes are needed to increase children and parents’ awareness of the health risks arising from food intakes deviating importantly from the recommendations. It should be further investigated in more detail how this preschool dietary pattern, which is in conflict with the Flemish FBDG, influences their nutrient intakes in order to check whether the current FBDG represent the most optimal dietary intake for this group of preschool-aged children. At last, research should assess the health risks associated with these unhealthy eating habits of young children, deviating importantly from the age specific recommendations.

## Figures and Tables

**Table 1:** Food intake calculated from estimated diet records, with adjustment for within-variability.

		**FBDG**	**Mean (SD)**				**Median (SE)**				**P**^¶¶^
			boys		girls		boys		girls		
Beverages*	< 4 y ≥ 4 y	1000 mL	495.2 542.8	(196.5) (243.3)	557.8 537.0	(193.4) (237.8)	434.0 513.0	(53.8) (41.8)	548.0 512.0	(70.4) (42.5)	0.603 0.763
Bread & cereals	< 4 y ≥ 4 y	90–150 g	104.9 99.6	(49.4) (31.1)	88.3 91.1	(28.5) (28.1)	96.0 97.0	(6.1) (2.5)	86.0 89.0	(3.7) (2.3)	0.004 0.002
Potatoes & grains (no crisps)	< 4 y ≥ 4 y	50–200 g	107.1 107.5	(26.6) (27.7)	99.8 99.1	(42.4) (25.3)	105.0 105.0	(3.3) (2.3)	93.0 97.0	(5.5) (2.1)	0.154 0.001
Vegetables (no juices & soups)	< 4 y ≥ 4 y	100–150 g	69.5 75.4	(18.5) (28.3)	61.5 73.3	(20.1) (31.7)	69.0 72.0	(2.3) (2.3)	60.0 71.0	(2.6) (2.6)	0.004 0.450
Fruit (no juices)	< 4 y ≥ 4 y	125–250 g	116.8 118.0	(63.1) (70.9)	121.3 107.2	(52.7) (50.0)	113.0 106.0	(7.8) (5.8)	119.0 105.0	(6.8) (4.2)	0.585 0.058
Milk products^μ^	< 4 y ≥ 4 y	500 mL	544.2 469.2	(240.6) (193.0)	483.1 425.0	(213.7) (198.1)	528.0 457.0	(29.9) (15.7)	475.0 401.0	(27.5) (16.4)	0.060 0.015
Cheese	< 4 y ≥ 4 y	10–20 g	11.0 14.2	(9.4) (11.2)	17.1 12.6	(11.8) (9.1)	10.3 12.9	(1.2) (0.9)	17.6 11.8	(1.5) (0.8)	<0.001 0.089
Meat, game, poultry and fish	< 4 y ≥ 4 y	75–100 g	90.3 99.7	(17.8) (26.3)	86.2 81.0	(24.1) (22.8)	89.0 97.0	(2.2) (2.1)	84.0 78.0	(3.1) (1.9)	0.174 <0.001
Spreadable fat	< 4 y ≥ 4 y	15–25 g	5.7 5.8	(6.3) (6.0)	3.8 4.7	(4.3) (5.1)	4.2 4.3	(0.8) (0.5)	2.3 3.0	(0.5) (0.4)	0.001 0.009
Restgroup (snacks/deserts)^†^	< 4 y ≥ 4 y	restricted	46.1 52.1	(13.7) (18.7)	47.1 55.3	(11.8) (12.0)	44.2 49.0	(1.7) (1.5)	45.9 54.5	(1.5) (1.0)	0.589 0.028
Restgroup (sugared drinks)^‡^	< 4 y ≥ 4 y	restricted	114.4 140.5	(121.0) (139.3)	62.3 110.1	(80.9) (124.3)	81.0 88.0	(15.0) (11.4)	33.0 60.0	(10.4) (10.3)	<0.001 0.013
Restgroup (fried potatoes)	< 4 y ≥ 4 y	restricted	12.7 13.9	(9.2) (6.8)	12.3 14.5	(9.4) (4.1)	11.4 12.9	(1.1) (0.6)	11.4 14.1	(1.2) (0.3)	0.735 0.233
Restgroup (sauces)	< 4 y ≥ 4 y	restricted	14.2 12.4	(7.3) (5.2)	13.3 12.9	(6.9) (11.8)	13.7 12.8	(0.9) (0.4)	12.8 11.8	(0.9) (1.0)	0.356 0.573
Restgroup (sweet spreads)^#^	< 4 y ≥ 4 y	restricted	16.4 14.5	(9.4) (9.4)	12.3 14.2	(7.3) (7.7)	14.4 12.4	(1.2) (0.8)	11.1 14.0	(0.9) (0.6)	<0.001 0.697

FBDG food-based dietary guidelines

* All drinks (incl. juices, but no milk products and no drinks from restgroup)

μ Milk, sugared milk drinks, yoghurt, milk deserts & calcium enriched soy drinks

† Restgroup - sweet deserts (e.g. ice cream, chocolate mousse), sweet snacks, salty snacks (e.g. chips), chocolate and brioches.

‡ Restgroup - sugared drinks (e.g. tea with sugar added) and soft drinks, but no juices.

# Restgroup - sweet spreads (e.g. chocolate spread, jam, etc.)

¶¶ Significance level of the differences between boys and girls according to t-test or Mann-Whitney U-test n (boys <4 years) 102 n (boys ≥4 years) 236 n (girls <4years) 95 n (girls ≥4years) 228

**Table 2:** Percentage of children below the lower limit and above the upper limit of the specific food-based dietary guideline.

			**< FBDG_LL_ (SE^¶^)**				**> FBDG_UL_ (SE^¶^)**			
			boys		girls		Boys		girls	
Beverages*	< 4 y ≥ 4 y	1000 mL	98.6 95.3	(1.2) (1.4)	98.3 95.9	(1.3) (1.3)	NA NA		NA NA	
Bread & cereals	< 4 y ≥ 4 y	90–150 g	43.3 40.4	(5.3) (4.0)	55.4 51.5	(5.7) (4.3)	14.4 6.2	(5.3) (2.8)	2.7 2.8	(2.5) (1.9)
Potatoes & grains (no crisps)	< 4 y ≥ 4 y	50–200 g	0.8 0.7	(0.9) (0.5)	8.6 0.8	(2.9) (0.6)	0.1 0.3	(0.3) (0.4)	2.6 0.1	(1.6) (0.2)
Vegetables (no juices & soups)	< 4 y ≥ 4 y	100–150 g	94.5 82.4	(2.3) (2.5)	96.1 82.1	(2.0) (2.5)	0.0 1.6	(0.8)	0.0 1.7	(0.9)
Fruit (no juices)	< 4 y ≥ 4 y	125–250 g	58.1 61.2	(4.9) (3.2)	54.6 66.4	(5.1) (3.1)	2.9 5.3	(1.7) (1.5)	1.3 0.8	(1.2) (0.6)
Milk products^μ^	< 4 y ≥ 4 y	500 mL	45.2 58.8	(4.9) (3.2)	54.9 68.1	(5.1) (3.1)	NA NA		NA NA	
Cheese	< 4 y ≥ 4 y	10–20 g	49.1 40.1	(4.9) (3.2)	29.1 41.7	(4.7) (3.3)	17.8 27.6	(3.8) (2.9)	41.8 19.3	(5.1) (2.6)
Meat, game, poultry and fish	< 4 y ≥ 4 y	75–100 g	19.9 17.4	(4.0) (2.5)	34.3 43.6	(4.9) (3.3)	28.0 46.1	(4.4) (3.2)	26.0 18.8	(4.5) (2.6)
Spreadable fat	< 4 y ≥ 4 y	15–25 g	91.0 92.0	(2.8) (1.8)	99.0 96.0	(1.0) (1.3)	1.0 1.0	(1.0) (0.6)	0.0 0.0	
Restgroup (snacks/deserts)^†^	< 4 y ≥ 4 y	restricted	NA NA		NA NA		33.7 48.6	(4.7) (3.3)	36.5 65.4	(4.9) (3.2)
Restgroup (sugared drinks)^‡^	< 4 y ≥ 4 y	restricted	NA NA		NA NA		44.0 45.5	(4.9) (3.2)	23.7 34.8	(4.4) (3.2)
Restgroup (fried potatoes)^η^	< 4 y ≥ 4 y	restricted	NA NA		NA NA		35.5 34.5	(4.7) (3.1)	35.7 41.0	(4.9) (3.3)
Restgroup (sauces)^§^	< 4 y ≥ 4 y	restricted	NA NA		NA NA		42.9 28.8	(4.9) (2.9)	20.7 13.7	(4.2) (2.3)
Restgroup (sweet spreads)^#^	< 4 y ≥ 4 y	restricted	NA NA		NA NA		47.3 38.3	(4.9) (3.2)	28.5 44.0	(4.6) (3.3)

FBDG_LL,_ food-based dietary guidelines Lower Level

FBDG_UL,_ food-based dietary guidelines Upper Level

* All drinks (incl. juices, but no milk products and no drinks from restgroup)

μ Milk, sugared milk drinks, yoghurt, milk deserts & calcium enriched soy drinks

† Restgroup - sweet deserts (e.g. ice cream, chocolate mousse), sweet snacks, salty snacks (e.g. chips), chocolate and brioches. Although the recommendation is to limit these food products, the percentage given in the column > FBDG_UL_ are children consuming more than 50 g/d of these snacks.

‡ Restgroup - sugared drinks (e.g. tea with sugar added) and soft drinks, but no juices. Although the recommendation is to limit these food products, the percentage given in the column > FBDG_UL_ are children consuming more than 100 ml/d of these sugared drinks.

η Restgroup - fried potatoes: although the recommendation is to limit these food products, the percentage given in the column > FBDG_UL_ are children consuming more than 15 g/d of fried potatoes.

§ Restgroup - sauces: although the recommendation is to limit these food products, the percentage given in the column > FBDG_UL_ are children consuming more than 15 g/d of sauces.

# Restgroup - sweet spreads (e.g. chocolate spread, jam, etc.): although the recommendation is to limit these food products, the percentage given in the column > FBDG_UL_ are children consuming more than 15 g/d of these sweet spreads.

¶ Standard error (SD) not displayed when percentage is 0 or 100

¶¶ Significance level of the differences between boys and girls according to t-test or Mann-Whitney U-test n (boys <4 years) 102 n (boys ≥4 years) 236 n (girls <4years) 95 n (girls ≥4years) 228

**Appendix 1:**
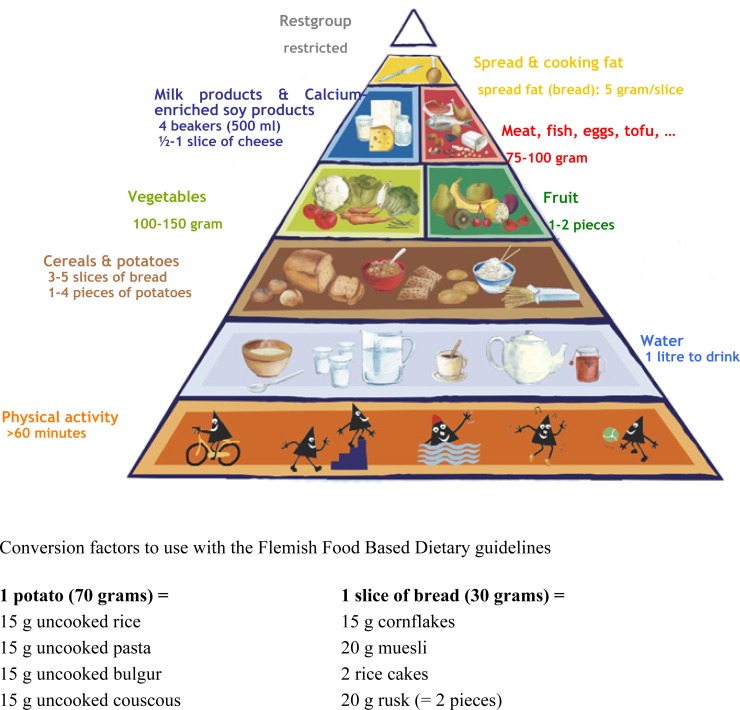
Flemish Food Based Dietary Guidelines: ‘the food triangle’. SOURCE: Adapted from the Flemish Institute for Health Promotion, Brussels, Belgium, www.vig.be

## Appendix 2: Food group categories used within the analyses

**Total beverages (incl. juices, no drinks from restgroup)**
Water
Light beverages
Tea and coffee without sugar
Fruit juice
Vegetable juice
Clear soup

**Bread and cereals**
Bread / rolls / crackers / rice cakes
Sugared bread
Breakfast cereals (ready-to-eat / hot)

**Potatoes and grains**
Pasta / noodles
Rice
Potatoes
Other grains / grain products^a^

**Vegetables**
Cooked vegetables
Raw vegetables

**Fruits (sweetened / unsweetened)**
Fresh fruit
Canned fruit
Dried fruit
Olives

**Milk, milk products and calcium enriched soy milk**
Milk^b^
Sugared milk drinks (e.g. fristi, chocolate milk, …)
Yoghurt
Sugared or aromatised yoghurt
Soy drinks
Milk desserts
Desserts on the basis of soy
Probiotics (e.g. actimel, yakult, …)
White (fresh) cheese

**Cheese**
Hard cheese
Cheese spread

**Meat / poultry / fish / egg / meat alternates**
Meat, game and meat products
Chicken / turkey
Fish / shellfish
Cold cuts (from meat poducts)
Cold cuts (from fish products)
Eggs^c^
Meat substitutes (e.g. tofu, tempe, …)
Nuts and seeds

**Fat & oil**
Butter / margarine
Oil
Frying oil

**Restgroup (snacks & desserts)**
Brioches
Sweet snacks
Salty snacks
Tea and coffee with sugar
Soft drinks
Salty sauces
Cream
Sweet sauces
Chocolate
Chocolate spread
Other sweet spread (e.g. jam, honey, …)
Sugar
Fried snacks
French fries / croquettes
Sweet desserts (e.g. ice cream, tiramisu, …)

**Miscellaneous^d^**

Food group classifications.

Food classification scheme was based on the food group classification of the Flemish food triangle. Food groups are based on 936 individual foods and include those consumed individually or as part of food mixtures that were disaggregated (spaghetti and other pasta-based dishes/casseroles/salads). However, some ingredients that were described as a recipe, but classified in the Flemish food triangle as one food were aggregated (e.g. the ingredients from bread were aggregated as bread, since bread was classified in the food triangle).

Notes: a Includes buckwheat groats/barley/couscous/quinoa/bran/wheat germ. b Includes cow’s milk and goat’s milk. c Includes only eggs reported separately and eggs included in disaggregated food mixtures. d Includes salt from disaggregated food mixtures /herbs and spices/monosodium glutamate/starch/plain gelatin /artificial sweeteners/pectin/cocoa powder.
